# Comparison of Modified Oblique Lateral Interbody Fusion and Posterior‐Only Approach in the Treatment of Degenerative Lumbar Scoliosis

**DOI:** 10.1111/os.70038

**Published:** 2025-04-07

**Authors:** Xiang Zhang, Yongqiang Wang, Yilin Lu, Junyu Li, Zhuoran Sun, Yan Zeng, Weishi Li, Miao Yu

**Affiliations:** ^1^ Department of Orthopedics Peking University Third Hospital Beijing China; ^2^ Beijing Key Laboratory of Spinal Disease Research Peking University Third Hospital Beijing China; ^3^ Engineering Research Center of Bone and Joint Precision Medicine Ministry of Education, Peking University Third Hospital Beijing China

**Keywords:** degenerative lumbar scoliosis, minimally invasive surgery, oblique lumbar interbody fusion, Wiltse approach

## Abstract

**Objective:**

Degenerative lumbar scoliosis (DLS) often requires surgical intervention, but traditional posterior‐only approaches, despite their effectiveness, result in significant muscle damage and high complication rates. Minimally invasive techniques like oblique lumbar interbody fusion (OLIF) and the Wiltse approach are preferred for preserving posterior structures. However, the lack of controlled studies comparing combined approaches to traditional methods limits their efficacy evaluation. The purpose of this study is to explore the clinical and radiological outcomes of OLIF with posterior fixation through Wiltse approach versus a posterior‐only approach in treating DLS.

**Methods:**

This retrospective study included 88 DLS patients underwent surgery from January 2019 to September 2021. The patients were divided into the OLIF group (*n* = 32) and the posterior group (*n* = 56). Comprehensive evaluations of clinical and radiological outcomes, including Cobb angle, coronal balance distance (CBD), sagittal vertical axis (SVA), thoracic kyphosis (TK), lumbar lordosis (LL), pelvic incidence (PI), pelvic tilt (PT), and sacral slope (SS) were conducted, with a subsequent subgrouping of OLIF group based on preoperative sagittal vertical axis (SVA) into Subgroup A (SVA ≤ 50 mm) and Subgroup B (SVA > 50 mm) for further analysis. The t‐test or Wilcoxon's rank sum test is used to compare continuous variables, and the chi‐square test is used to compare categorical variables.

**Results:**

The OLIF group had fewer fixation levels (4.25 ± 1.08 vs. 5.56 ± 2.04, *p* < 0.001) and shorter hospitalization (5.22 ± 2.25 d vs. 6.66 ± 2.16 d, *p* < 0.001), fewer drainage volume (371.94 mL vs. 1065.25 mL, *p* < 0.001), but longer surgical time. Postoperatively, the OLIF group showed better clinical outcomes. In both groups, Cobb angle, coronal balance distance, and sagittal spinal pelvic parameters improved significantly. The OLIF group achieved a lower SVA (23.84 mm ± 36.70 mm vs. 42.84 mm ± 36.25 mm, *p* = 0.027), which was not maintained at the final follow‐up. Subgroup A maintained sagittal balance (34.55 mm ± 24.99 mm vs. 83.73 mm ± 61.90 mm, *p* = 0.029). Moreover, the OLIF group had fewer complications.

**Conclusion:**

Minimally invasive multi‐level OLIF with posterior fixation through Wiltse approach, as compared to the conventional posterior approach, has fewer fixation segments, offers comparable radiographic outcomes and, more importantly, superior clinical results. In addition, patients with a preoperative SVA > 50 mm could benefit from more fixation levels to maintain sagittal balance.

## Graphical abstract

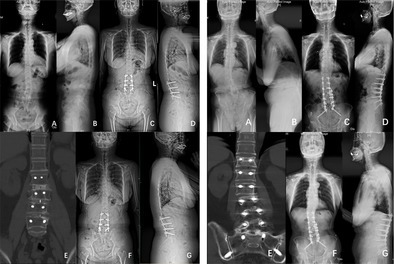

## 1 Introduction

Degenerative lumbar scoliosis (DLS) is characterized by coronal scoliosis with a Cobb angle exceeding 10° [1]. Surgical intervention is typically necessary to alleviate symptoms, correct both coronal and sagittal deformities, and restore spinal balance [2]. The traditional posterior‐only approach, involving various osteotomies, has been a robust surgical technique for treating spinal deformities [3]. However, its damage to the paravertebral muscles can compromise postoperative clinical outcomes, with an overall complication rate ranging from 40% to 86% [4]. This risk is particularly noteworthy for DLS patients with comorbidities requiring long‐segment fixation to maintain sagittal balance [5, 6].

Consequently, minimally invasive surgeries (MIS) have gained popularity among elderly patients in recent years, including oblique lumbar interbody fusion (OLIF), anterior lumbar interbody fusion (ALIF), and extreme/direct lateral interbody fusion (D/XLIF). OLIF is carried out through an anterolateral approach and is a variety of lateral lumbar interbody fusion (LLIF). Compared with the posterior surgery, the lateral approach preserves the stable structure of the posterior column and reduces soft tissue damage, which can maintain lumbar spine stability as much as possible. With the implantation of lordotic cages, both coronal and sagittal alignments can be corrected sufficiently [7, 8, 9]. The Wiltse approach is a muscle‐sparing technique that goes through the sacrospinalis muscle and between the multifidus and longissimus, it has less risk of wound breakdown, infection, and blood loss compared to the midline approach.

The lateral approach bone fusion has been reported to yield significant outcomes in the treatment of single‐segment lumbar degenerative diseases. However, there have been only a limited number of studies exploring its feasibility for mild to moderate DLS, and these studies lack control groups for comparison [8, 9, 10, 11]. In addition, percutaneous screw fixation has traditionally been used in OLIF surgery. However, long incisions and difficulty in positioning have hindered the application of percutaneous screws in spinal orthopedic surgery. Therefore, we have improved the OLIF technique and adopted the Wiltse approach for internal fixation instead of percutaneous screws. The purposes of the study are as follows: (i) to compare the clinical and radiographic outcomes of a combined approach (LLIF with posterior Wiltse approach fixation) with the traditional posterior‐only approach; (ii) to propose potential fusion level selection strategies for the combined approach.

## 2 Materials and Methods

### 2.1 Patient Selection

**FIGURE 1:**
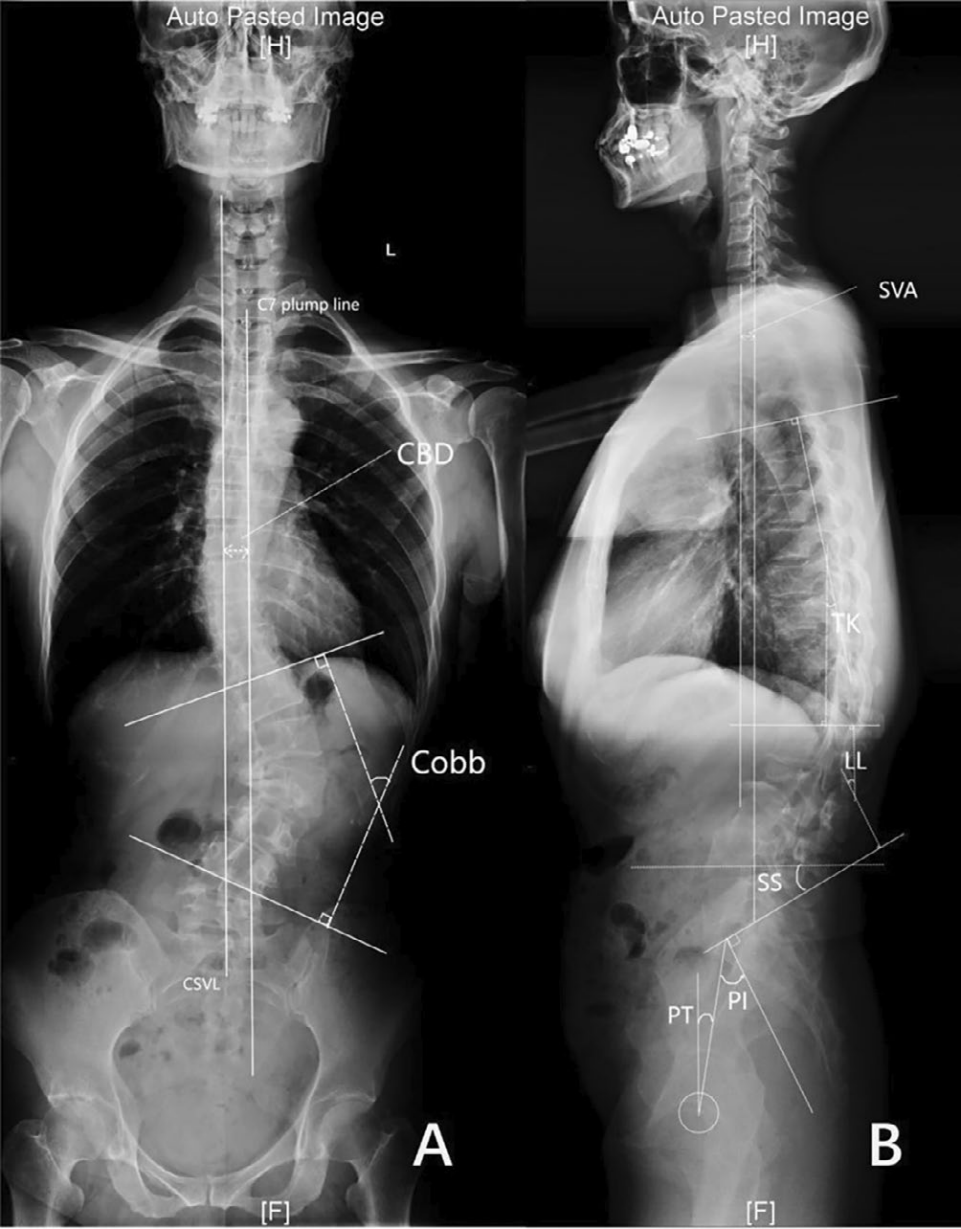
Imaging parameter measurement diagram.

This retrospective study was approved by the Institutional Review Board of Peking University Third Hospital (M20250179) and all patients agreed to participate in the study. A total of 88 DLS patients who underwent surgery between January 2019 and September 2021 and had available preoperative and postoperative radiographs were included (Figure 1). Inclusion criteria were as follows: (1) patients aged ≥ 55 years experiencing symptomatic back and/or leg pain attributable to adult spinal deformity (ASD); (2) patients exhibiting a Cobb angle > 10° or sagittal vertical axis (SVA) > 50 mm; (3) patients with a minimum follow‐up period of 24 months post‐surgery. Exclusion criteria encompassed: (1) a history of prior lumbar surgery, spinal trauma, or hip surgery; (2) kyphosis resulting from traumatic, osteoporotic compression fractures, or infection.

Patients were divided into two groups based on the surgical technique employed: (1) the OLIF group (*n* = 32), which underwent a lateral approach with interbody fusion combined with pedicle screw fixation via the Wiltse approach; (2) the posterior‐only group (*n* = 56), which underwent osteotomy, lumbar interbody fusion, and pedicle screw fixation via a midline approach.

### 2.2 Operative Technique

#### 2.2.1 OLIF Group

Patients were placed in a lateral decubitus position. A transverse skin incision was performed on the convex side. The abdominal muscle layers were separated bluntly, and the anterior edge of the psoas was identified. A circumferential retractor was placed using a lateral retropleural or retroperitoneal approach to protect vessels. The intervertebral disc, along with the cartilaginous endplates, was completely excised following localization. Subsequently, an appropriately lordotic cage filled with allograft bone was inserted into the intervertebral space. The patient was then repositioned in a prone position to undergo posterior internal fixation through the Wiltse approach.

#### 2.2.2 Posterior Group

Patients were placed in a prone position, and a midline incision was conducted to expose the posterior elements of the target segments. Erector spinae retraction allowed for the exposure of the laminae, spinous processes, and facet joints. Pedicle screws were inserted bilaterally. Different osteotomy and various lumbar interbody fusion (TLIF or PLIF) techniques were applied depending on the curve's characteristics.

### 2.3 Clinical and Radiologic Evaluation

Data collected included age, gender, body mass index (BMI), *T*‐score of bone mineral density (BMD), follow‐up time, Lenke‐Silva classification, surgical details, and clinical and radiological results. Surgical details involved techniques used, fixation levels, fusion levels, operation time, estimated blood loss (EBL), drainage volume, length of hospital stay, and complications. Back and leg pain was measured with the visual analogue scale (VAS), Oswestry Disability Index (ODI), and Japanese Orthopedic Association (JOA) score both preoperatively and at the final follow‐up. Cobb angle, CBD, SVA, TK, LL, PI, PT, and SS were measured [12]. All digital radiographs were evaluated by two authors independently. The inconsistent results were measured again by a third, more senior surgeon. These doctors were only responsible for the measurements and were not aware of the study.

### 2.4 Statistical Analysis

Continuous variables were analyzed with an unpaired *t*‐test if they demonstrated a normal distribution, otherwise Wilcoxon's rank‐sum test was used. Categorical variables were evaluated with a chi‐square test. Our analysis was conducted using IBM SPSS Statistics 26 (IBM Corporation, Armonk, NY, USA), and any *p* value lower than 0.05 was deemed statistically significant.

## 3 Results

### 3.1 Patient Demographics and Surgical Characteristics

A total of 88 patients were included in this study, comprising five males and 83 females (Figure 1). The OLIF group consisted of 32 patients, and the posterior group had 56 patients. The two groups showed no significant differences in age, gender, height, weight, BMI, *T*‐score of BMD, Lenke‐Silva classification, and postoperative follow‐up time.

The OLIF group demonstrated significantly lower fixation levels (4.25 ± 1.08 vs. 5.56 ± 2.04, *p* = 0.004), shorter hospitalization durations (5.22 ± 2.25 d vs. 6.66 ± 2.16 d, *p* < 0.001), and reduced drainage volumes (371.94 ± 231.67 mL vs. 1065.25 ± 559.43 mL, *p* < 0.001) compared to the posterior group. However, the OLIF group required a longer surgical time (450.88 ± 99.47 min vs. 262.57 ± 76.92 min, *p* < 0.001). In addition, the EBL was lower in the OLIF group compared with the posterior group (608.71 ± 280.15 mL vs. 760.54 ± 461.40 mL, *p* = 0.099), although the difference was not statistically significant (Table 1).

**TABLE 1:** Demographics and surgical characteristics between two groups.

Variables	OLIF group (*n* = 32)	Posterior group (*n* = 56)	*p*
Mean age (years)	66.25 ± 6.78	65.93 ± 6.33	0.824
Female (%)	90.91	92.86	0.742
Height (m)	1.57 ± 0.06	1.58 ± 0.07	0.584
Weight (kg)	63.59 ± 7.61	64.5 ± 9.72	0.651
BMI (kg/m^2^)	25.86 ± 2.64	26.02 ± 3.95	0.844
BMD (*T*‐score)	−2.32 ± 0.87	−2.56 ± 0.91	0.361
Follow‐up (month)	32.84 ± 5.13	33.52 ± 7.52	0.620
Operation time (min)	450.88 ± 99.47	262.57 ± 76.92	< 0.001
EBL (ml)	608.71 ± 280.15	760.54 ± 461.4	0.099
Hospitalization (day)	5.22 ± 2.25	6.66 ± 2.16	0.004
Drainage volume (ml)	371.94 ± 231.67	1065.25 ± 559.43	< 0.001
Fixation levels	4.25 ± 1.08	5.56 ± 2.04	< 0.001
Lenke‐Silva levels			
Level I	0	0	0.780
Level II	7	13	
Level III	7	7	
Level IV	3	4	
Level V	14	29	
Level VI	1	3	

### 3.2 Clinical Outcomes

No significant difference was observed in preoperative demographic data or VAS (lumbar), VAS (leg), ODI, and JOA between the two groups (*p* = 0.914, 0.269, 0.854, 0.144, respectively). At the final follow‐up, significant improvements were seen in all clinical parameters for the OLIF group compared to the preoperative period (all *p <* 0.001). The OLIF group had a lower ODI score (4.26 ± 1.26 vs. 5.68 ± 3.40, *p* = 0.028) and a higher JOA score (24.94 ± 2.23 vs. 23.34 ± 3.32, *p* = 0.019) compared to the posterior group. VAS (lumbar) and VAS (leg) in the final follow‐up were not significantly different between the two groups (1.29 ± 1.51 vs. 1.50 ± 1.91, *p* = 0.599; 0.13 ± 0.43 vs. 0.27 ± 0.70, *p* = 0.255) (Table 2).

**TABLE 2:** Clinical parameters between two groups.

Variables	OLIF group (*n* = 32)	Posterior group (*n* = 56)	*p*
VAS (lumbar)			
Preoperation	5.94 ± 1.83	5.89 ± 1.87	0.914
Final follow up	1.29 ± 1.51	1.50 ± 1.91	0.599
*p* value (pre‐final)	< 0.001	< 0.001	
VAS (leg)			
Preoperation	3.59 ± 2.80	4.3 ± 2.92	0.269
Final follow up	0.13 ± 0.43	0.27 ± 0.70	0.255
*p* value (pre‐final)	< 0.001	< 0.001	
ODI			
Preoperation	23.41 ± 7.87	23.04 ± 9.67	0.854
Final follow up	4.26 ± 1.26	5.68 ± 3.40	0.028
*p* value (pre‐final)	< 0.001	< 0.001	
JOA			
Preoperation	17.63 ± 7.67	15.54 ± 5.55	0.144
Final follow up	24.94 ± 2.23	23.34 ± 3.32	0.019
*p* value (pre‐final)	< 0.001	< 0.001	

### 3.3 Radiographic Outcomes

The radiographic outcomes are shown in Table 3. There was no statistically significant difference in Cobb, CBD, PI, PT, SS, TK, LL, PI‐LL mismatch, and SVA between the OLIF and posterior groups preoperatively (all *p* > 0.005). Postoperatively, the SVA was significantly lower in the OLIF group (23.84 ± 36.70 mm vs. 42.84 ± 36.25 mmm, *p* = 0.027). Other parameters improved in both groups after surgery, but there was no statistically significant difference between groups (all *p* > 0.005). At the final follow‐up, significant improvements were seen in Cobb angle, TK, LL, PT, and PI‐LL mismatch in both groups compared to preoperative data (all *p* < 0.005). However, only the posterior group showed statistical differences in SVA (73.08 ± 49.28 mm vs. 42.84 ± 36.25 mm, *p* = 0.027) compared to the preoperative period. While the SVA in the OLIF group was not maintained until the final follow‐up, it was larger than the posterior group (58.06 ± 51.81 mm vs. 44.75 ± 33.27 mm, *p* = 0.200).

**TABLE 3:** Radiographic parameters between OLIF group and posterior group.

Variables	OLIF group (*n* = 32)	Posterior group (*n* = 56)	*p*
Cobb (°)			
Preoperation	29.62 ± 13.69	28.32 ± 10.14	0.643
Postoperation	8.52 ± 4.73	8.81 ± 4.77	0.795
Final follow up	8.05 ± 5.71	8.1 ± 4.71	0.973
*p* value (pre‐final)	< 0.001	< 0.001	
CBD (mm)			
Preoperation	15.70 ± 22.74	17.42 ± 15.85	0.677
Postoperation	15.45 ± 13.35	30.15 ± 73.82	0.301
Final follow up	11.22 ± 15.42	15.81 ± 12.74	0.194
*p* value (pre‐final)	0.393	0.340	
PI (°)			
Preoperation	51.56 ± 15.00	52.39 ± 14.13	0.796
Postoperation	47.21 ± 12.08	50.65 ± 10.78	0.196
Final follow up	49.70 ± 11.29	49.88 ± 11.21	0.951
*p* value (pre‐final)	0.202	0.104	
PT (°)			
Preoperation	30.85 ± 12.62	30.83 ± 10.82	0.994
Postoperation	23.70 ± 11.87	24.63 ± 9.33	0.700
Final follow up	25.16 ± 11.78	25.92 ± 10.24	0.783
*p* value (pre‐final)	0.008	< 0.001	
SS (°)			
Preoperation	20.74 ± 12.10	22.7 ± 10.96	0.440
Postoperation	24.49 ± 8.26	26.32 ± 9.38	0.398
Final follow up	25.07 ± 6.86	24.32 ± 10.56	0.759
*p* value (pre‐final)	0.234	0.350	
TK (°)			
Preoperation	21.81 ± 15.81	20.84 ± 11.78	0.745
Postoperation	25.73 ± 11.68	24.60 ± 10.20	0.653
Final follow up	28.40 ± 12.19	26.59 ± 14.54	0.609
*p* value (pre‐final)	0.009	0.012	
LL (°)			
Preoperation	24.36 ± 18.75	24.88 ± 16.25	0.891
Postoperation	32.19 ± 3.01	34.99 ± 12.2	0.337
Final follow up	32.17 ± 14.11	32.55 ± 13.89	0.916
*p* value (pre‐final)	0.004	0.001	
PI‐LL mismatch (°)			
Preoperation	27.20 ± 20.73	27.51 ± 15.46	0.937
Postoperation	13.33 ± 19.57	15.66 ± 13.61	0.529
Final follow up	17.52 ± 17.63	17.33 ± 15.18	0.963
*p* value (pre‐final)	0.002	< 0.001	
SVA (mm)			
Preoperation	62.78 ± 48.06	73.08 ± 49.28	0.314
Postoperation	23.84 ± 36.70	42.84 ± 36.25	0.027
Final follow up	58.06 ± 51.81	44.75 ± 33.27	0.200
*p* value (pre‐final)	0.263	< 0.001	

We further divided the OLIF group into Subgroup A (preoperative SVA ≤ 50 mm) and Subgroup B (preoperative SVA > 50 mm) (Table 4). Fixation levels did not significantly differ between these groups (4.22 ± 1.22 vs. 4.29 ± 0.91, *p* = 0.872). Postoperative SVA was 18.06 ± 38.06 mm for Subgroup A and 32.76 ± 34.26 mm for Subgroup B, with only Subgroup A maintaining the sagittal balance at the final follow‐up (34.55 ± 24.99 mm vs. 83.73 ± 61.90 mm, *p* = 0.029).

**TABLE 4:** Subgroup analysis between Subgroup A and Subgroup B.

Variables	Subgroup A (*n* = 18)	Subgroup B (*n* = 14)	*p*
Fixation level	4.22 ± 1.22	4.29 ± 0.91	0.872
SVA (mm)			
Preoperation	27.19 ± 13.07	108.54 ± 35.71	< 0.001
Postoperation	18.06 ± 38.06	32.76 ± 34.26	0.310
Final follow up	34.55 ± 24.99	83.73 ± 61.90	0.029

### 3.4 Complications

There were 11 complications in the OLIF group and 56 complications in the posterior group (Table 5). In the OLIF group, the most common perioperative complications were waist/leg weakness/numbness, gastrointestinal symptoms, and cerebrospinal fluid leaks (each occurring in 2/32 patients, 6.25%). Rare complications included wound infections and pulmonary embolism (1/32, 3.12%). Pulmonary embolism occurred on the third day postoperatively, prompting the implantation of an inferior vena cava filter, after which the patient's condition improved. Subsequently, the patient was transferred to the respiratory medicine department for anticoagulation therapy with heparin. In comparison, the posterior group's most common complication was waist/leg weakness/numbness (7/56, 12.5%), followed by reoperation (5/56, 8.93%), cerebrospinal fluid leaks (4/56, 7.14%), wound infections, hematomas, and pulmonary embolism (each occurring in 2/56 patients, 3.57%). The causes for reoperation were as follows: two instances of inadequate decompression, one instance of epidural hematoma, one instance of screw loosening, and one instance of rod breakage.

**TABLE 5:** Complications between OLIF group and posterior group.

Variables	OLIF group (*n* = 32)	Posterior group (*n* = 56)	*p*
Perioperative complication			
Waist/leg weakness/numbness	2 (6.25%)	7 (12.50%)	0.352
Reoperation	0 (0%)	5 (8.93%)	0.082
Cerebrospinal fluid leak	2 (6.25%)	4 (7.14%)	0.873
Wound infection	1 (3.12%)	2 (3.57%)	0.912
Hematoma	0 (0%)	2 (3.57%)	0.280
Pulmonary embolism	1 (3.12%)	2 (3.57%)	0.912
Gastrointestinal symptoms	2 (6.25%)	0 (0%)	0.130
Segmental artery injury	1 (3.12%)	0 (0%)	0.183
Sympathetic nerve injury	2 (6.25%)	0 (0%)	0.130
Late complication			
PJK	2 (6.25%)	6 (10.71%)	0.705
Screw loosening	0 (0%)	20 (35.71%)	< 0.001
Rod breakage	0 (0%)	1 (1.79%)	0.447
Cage subsidence	2 (6.25%)	3 (5.36%)	0.862
Pseudarthrosis	0 (0%)	4 (7.14%)	0.122
Total	14	56	

Late complications were predominantly seen in the posterior group, with the most common complication being screw loosening (20/56, 35.71%), followed by proximal junctional kyphosis (PJK) (6/56, 10.71%), pseudarthrosis (4/56, 7.14%), cage subsidence (3/56, 5.36%), and rod breakage (1/56, 1.79%). In the OLIF group, cage subsidence and PJK occurred in two patients, respectively. Significantly, more screw loosening was observed in the posterior group (*p* < 0.001).

**FIGURE 2:**
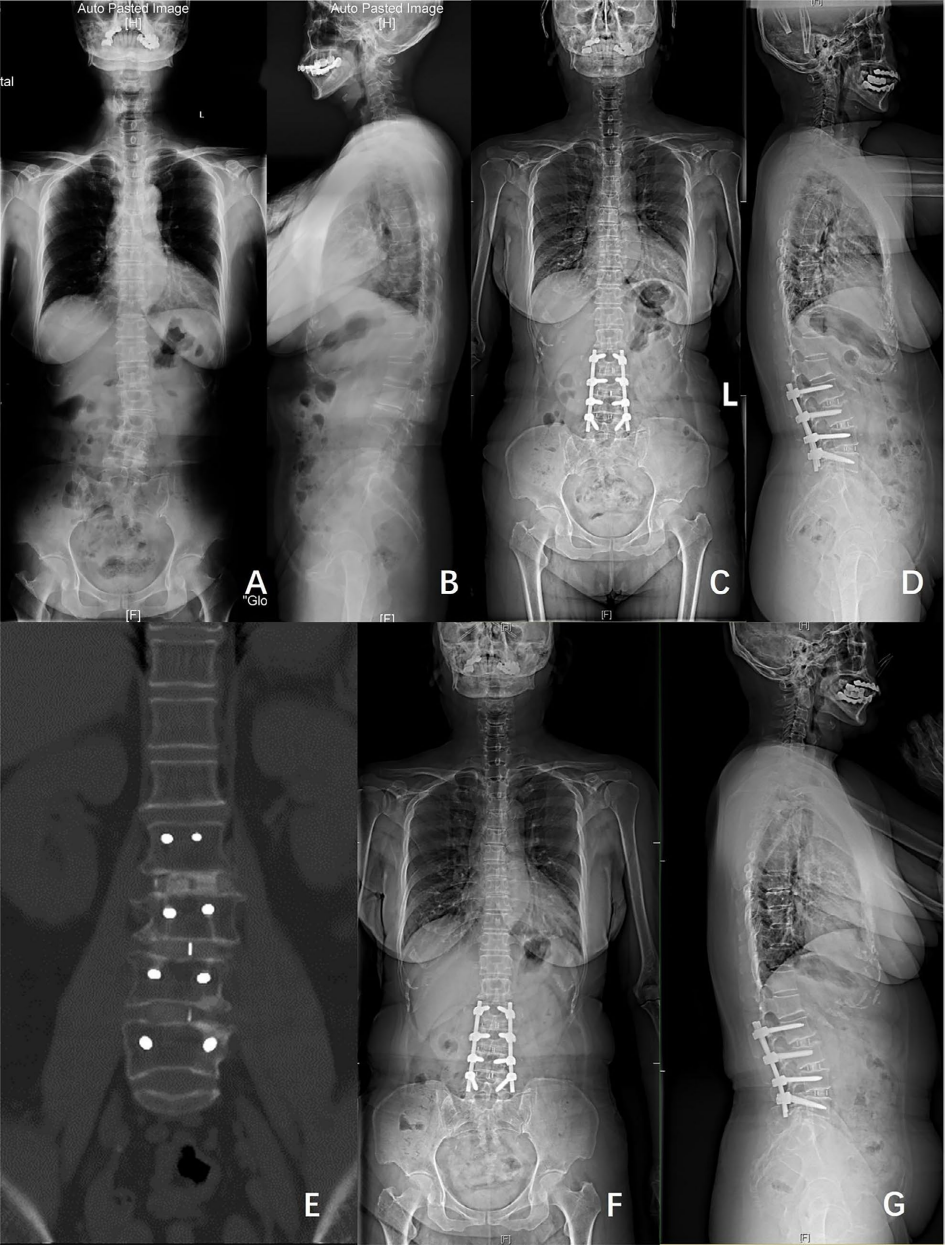
Case example of a 62‐year‐old female with DLS who underwent surgical correction consisting of L2–L5 OLIF with posterior instrumentation. (A and B) Full‐length standing posteroanterior and lateral radiographs before operation (Cobb 22.1°, CDB 11.21 mm, PI 38.3°, PT 20.8°, SS 18.2°, TK 16.5°, LL 4.0°, SVA 0 mm). (C and D) Posteroanterior and lateral standing radiographs 1 year after operation (Cobb 3.3°, CDB 11.72 mm, PI 31.0°, PT 13.5°, SS 18.9°, TK 21.6°, LL 26.9°, SVA 1.15 mm). (E) CT scan 1 year after surgery showed bone fusion at L2–L5. (F and G) Posteroanterior and lateral standing radiographs 2 years after operation (Cobb 2.6°, CDB 4.34 mm, PI 35.0°, PT 8.8°, SS 26.7°, TK 19.9°, LL 34.9°, SVA 11.0 mm).

**FIGURE 3:**
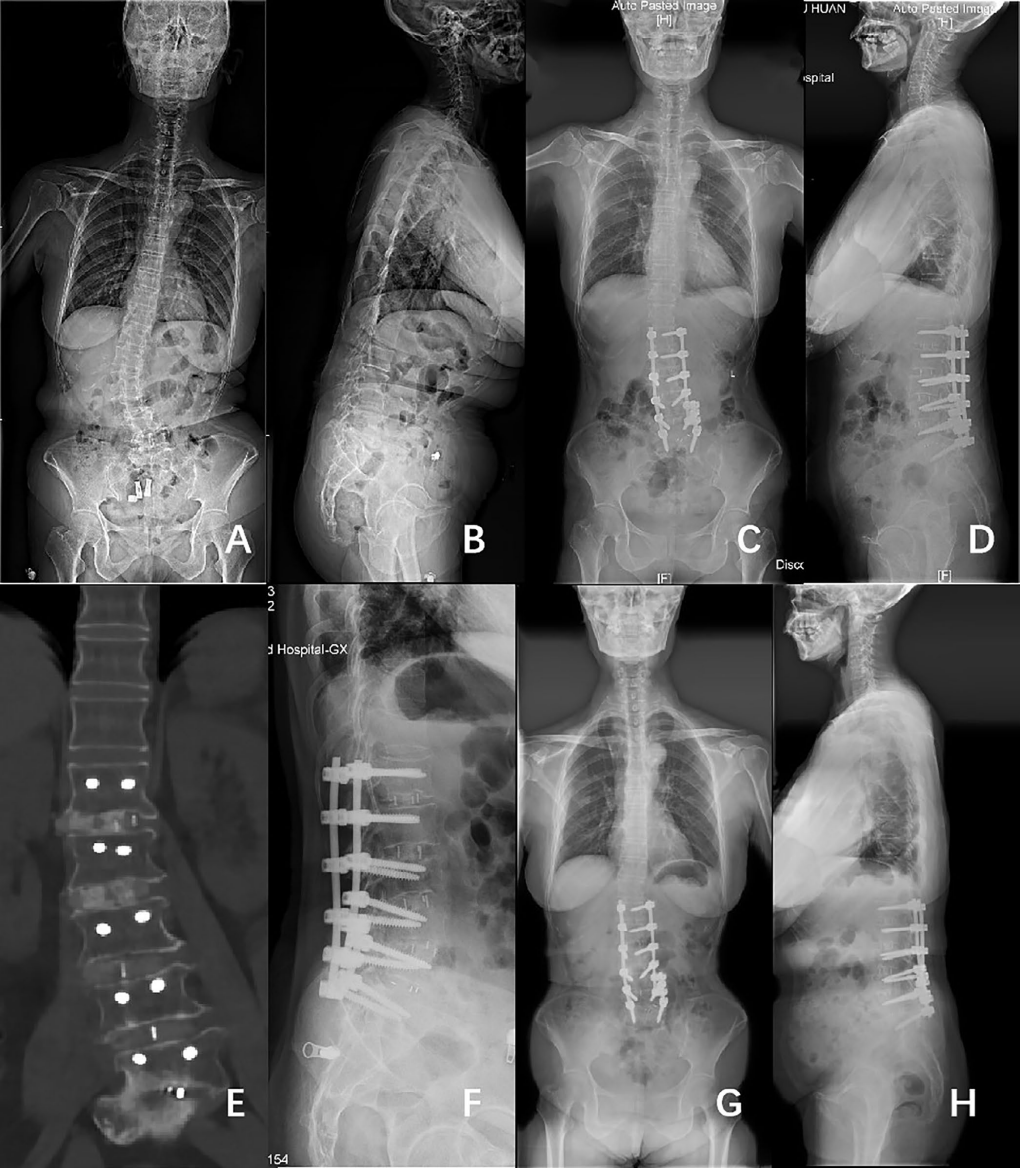
Case example of a 62‐year‐old female with DLS who underwent surgical correction consisting of L1–L5 OLIF with posterior instrumentation. (A and **B**) Full‐length standing posteroanterior and lateral radiographs before operation (Cobb 32.0°, CBD 19.84 mm, PI 58.3°, PT 36.3°, SS 22.1°, TK 2.9°, LL 1.5°, SVA 145.25 mm). (C and D) Posteroanterior and lateral standing radiographs 1 year after operation (Cobb 8.0°, CBD 13.51 mm, PI 58.3°, PT 35.8°, SS 22.8°, TK 15.5°, LL 26.8°, SVA 48.46 mm). (E) CT scan 1 year after surgery showed bone fusion at L1–L5. (F) Cage subsidence was observed 1 year after surgery. (G and H) Posteroanterior and lateral standing radiographs 2 years after operation (Cobb 8.6°, CBD 15.83 mm, PI 52.8°, PT 35.7°, SS 17.3°, TK 6.5°, LL 17.5°, SVA 51.84 mm).

Figures 2 and 3 show classical examples of OLIF and posterior‐only approach surgery, respectively.

## 4 Discussion

This retrospective evaluation demonstrates that minimally invasive multilevel OLIF combined with posterior fixation through the Wiltse approach, compared to the conventional posterior approach, achieves similar radiographic improvements at a minimum 2‐year follow‐up, while the OLIF with Wiltse approach group exhibits better clinical outcomes and fewer complications. However, additional fixation levels may be beneficial for patients with a preoperative SVA exceeding 50 mm to ensure optimal sagittal balance.

### 4.1 Clinical and Radiographic Outcomes Between Two Groups

In our study, both groups exhibited significant improvements in global coronal and sagittal balance based on radiologic findings. The Cobb angle was reduced significantly after surgery in both groups and remained stable at the final follow‐up, which was similar to the previous reports [7, 8]. Moreover, the CBD of both groups remained within 30 mm after surgery and at the final follow‐up. We also want to highlight the better clinical outcomes of patients in the OLIF group. OLIF surgery is mostly performed in the muscle space, causing less damage to the muscle and better preservation of muscle function. As a result, patients in the OLIF group had less disability at the last follow‐up.

Conventionally, posterior‐only approaches with different osteotomies or lumbar interbody fusion have been potent surgical techniques for spinal deformity treatment [3, 13]. However, patients with DLS often accompany comorbidities and require long‐segment fixation to maintain sagittal balance, which is associated with high risks of medical complications such as infection, neurologic complications, deep venous thrombosis, pulmonary embolism, dural tears, and so forth [4]. Mechanical complication rates reported in previous studies range from 20% to 50%, including issues like pseudarthrosis, PJK, screw loosening, rod breakage, and cage subsidence [14].

**FIGURE 4:**
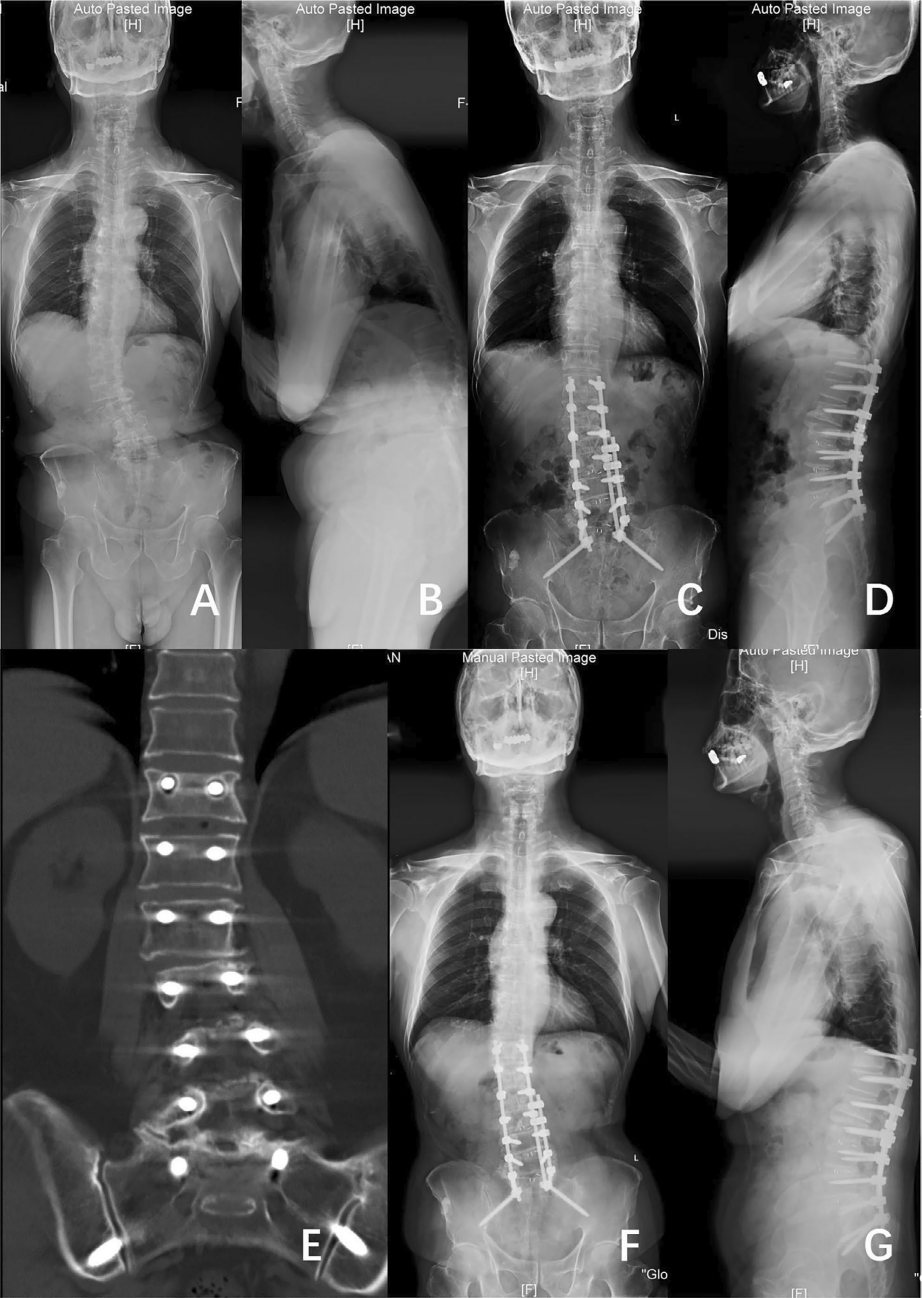
Case example of a 74‐year‐old male with DLS who underwent surgical correction consisting of T12‐S2AI posterior surgery. (A and B) Full‐length standing posteroanterior and lateral radiographs before operation (Cobb 18.6°, CDB 15.39 mm, PI 49.1°, PT 35.3°, SS 12.7°, TK 21.4°, LL 5.5°, SVA 173.56 mm). (C and D) Posteroanterior and lateral standing radiographs 1 year after operation (Cobb 5.5°, CDB 13.22 mm, PI 44.6°, PT 25.0°, SS 20.7°, TK 33.0°, LL 29.6°, SVA 15.82 mm). (E) T12 screw loosening was found 1 year after surgery by CT. (F and G) Posteroanterior and lateral standing radiographs 2 years after operation (Cobb 7.5°, CDB 10.06 mm, PI 47.7°, PT 28.4°, SS 15.6°, TK 29.9°, LL 26.1°, SVA 76.16 mm).

Silvestre et al. [15] proposed OLIF as a surgical approach that utilizes the space between the retroperitoneal aorta and the psoas major muscle to access intervertebral discs. Compared with the posterior approach, OLIF allows for multi‐segmental fusion without compromising the bony stabilizing structures of the posterior column in the spine, thus reducing soft tissue damage and minimizing the risk of neurological injuries. Meta‐analyses indicate that OLIF is safe and effective for treating mild to moderate ASD, which can reduce intraoperative blood loss and lower perioperative complications [9]. Moreover, previous studies have confirmed the effectiveness of OLIF combined with techniques such as posterior column osteotomy, anterior longitudinal ligament release, and anterior column realignment in treating DLS with severe sagittal imbalance [16, 17]. In our study, patients in both groups displayed consistent preoperative Lenke‐Silva classifications. The results suggest that OLIF could achieve similar radiological outcomes with a reduction in the occurrence of complications compared to the posterior‐only approach (Figure 4).

### 4.2 Optimal Fixation Levels in OLIF Surgery

Zhou et al. [18] examined age‐dependent normative values for sagittal parameters and established their interrelationships within the Chinese population. The study observed a significant age‐related increase in the T1 pelvic angle, suggesting that the degeneration of the thoracolumbar segment is the primary unit of curvature change during the aging process among Chinese individuals. Consequently, the correction of thoracolumbar deformity ranked first in our surgical strategy.

While the OLIF group showed a more significant SVA improvement immediately after surgery compared to the posterior group, it cannot be sustained at the final follow‐up. This was because the OLIF group had fewer fixation levels, thus less robust correction, than the posterior group. Previous studies have indicated that increasing the fixation levels, even extending to the pelvic, can facilitate SVA maintenance after OLIF for patients without sagittal balance [10, 13, 19]. Additionally, Yang et al. [11] report patients with a preoperative SVA < 50 mm have maintained the SVA postoperatively with fewer fixation levels. Therefore, we further divided the OLIF group based on preoperative SVA into two subgroups, which had similar fixation levels. Postoperative sagittal balance was achieved in both subgroups; however, Subgroup B did not maintain SVA at the final follow‐up. This outcome suggests that a preoperative SVA > 50 mm necessitates more fixation levels.

### 4.3 OLIF Has Less Complication Than Traditional Approach

The OLIF group experienced fewer complications than the posterior group. In the posterior group, five patients required reoperation, including two cases of incomplete decompression, one of hematoma formation, one of screw loosening, and one of rod breakage. Cerebrospinal fluid leaks occurred in the OLIF group during decompression. Notably, wound infection in the OLIF group appeared in the abdominal incision, and two patients experienced gastrointestinal symptoms, possibly due to the long time retraction of the intestine and wound to establish a surgical channel. The posterior group had more late complications. Studies have shown that the degeneration of the paraspinal muscle, the increase of fusion segment, pedicle subtraction osteotomy, and fixation to sacrum were risk factors for screw loosening and PJK in patients with adult degenerative scoliosis [19, 20, 21]. The occurrence of mechanical complications on reducing in the OLIF group may lie in referring to fewer fixation levels and carrying out the Wiltse approach, which maximizes the protection of the paraspinal muscles and spine motions.

### 4.4 Limitations and Strengths

There are a few limitations in our study. It is a single‐center, retrospective, and observational study with a small sample size, necessitating larger prospective studies for validation. Due to the small number of patients receiving MR at follow‐up, we lack effective methods for assessing paravertebral muscles. The strength of this study lies in its comprehensive comparison of clinical outcomes, radiographic results, and complication rates between the two surgical approaches, as well as its proposal of optimal fixation segment strategies for maintaining sagittal balance, supported by an analysis of data from previous studies on sagittal balance correction in DLS treated with OLIF.

## 5 Conclusion

Minimally invasive multilevel LLIF with pedicle screw fixation through the Wiltse approach can achieve similar radiographic outcomes as the traditional posterior approach, with better clinical results. The OLIF group experienced fewer complications, required fewer fixation levels, and patients experienced better subjective feelings. However, patients with a preoperative SVA > 50 mm could not maintain sagittal balance.

## Author Contributions

**Xiang Zhang:** data curation, formal analysis, investigation. **Yongqiang Wang:** conceptualization, project administration, writing – review and editing. **Yilin Lu:** conceptualization, formal analysis, methodology, writing – original draft. **Junyu Li:** visualization. **Zhuoran Sun:** methodology, writing – review and editing. **Yan Zeng:** data curation. **Weishi Li:** resources. **Miao Yu:** conceptualization, project administration, writing – review and editing.

## Conflicts of Interest

The authors declare no conflicts of interest.
